# Application of Bag-Controlled Release Fertilizer Facilitated New Root Formation, Delayed Leaf, and Root Senescence in Peach Trees and Improved Nitrogen Utilization Efficiency

**DOI:** 10.3389/fpls.2021.627313

**Published:** 2021-03-31

**Authors:** Yafei Zhang, Jingjing Luo, Futian Peng, Yuansong Xiao, Anqi Du

**Affiliations:** State Key Laboratory of Crop Biology, College of Horticulture Science and Engineering, Shandong Agricultural University, Tai-An, China

**Keywords:** bag-controlled release fertilizer, plant growth and senescence, ammonia volatilization, N utilization rate, storage nutrition, peach

## Abstract

It is very important to promote root growth and delay root and leaf senescence, to improve nitrogen absorption and utilization efficiency, and to improve the storage nutrition level of the tree, so as to improve the fruit quality and yield of peach. In this experiment, we compared and analyzed the effects of traditional fertilization and bag-controlled release fertilizer (BCRF) on the growth of shoots and roots, senescence of leaves and roots, and fruit yield and quality. Moreover, the impacts of BCRF on ammonia volatilization, nitrogen utilization rate, fine root turnover, and plant storage nutrients were also investigated. Compared with conventional fertilizer use, the application of BCRF significantly promoted the shoot growth of young peach trees. Additionally, BCRF delayed leaf senescence and increased root activity in autumn. This increased the storage nutrients of the peach tree. Compared with traditional fertilizer, ammonia volatilization reduced to 54.36% under BCRF application situation. BCRF also promoted the occurrence of fine roots and decreased the annual turnover rate. A ^15^N tracer test showed that, compared with traditional fertilizer, BCRF nitrogen utilization efficiency increased by 37.73% in peach trees under BCRF treatment significantly. The results from 3 consecutive years showed that the application of BCRF increased the yield of individual plants by 21.35% on average compared to the yield from plants receiving equal amounts of fertilizer applied by spreading (FSA). Thus, BCRF can promote the occurrence of fine roots and decrease the root annual turnover rate in peach trees, and it also improves the utilization efficiency of fertilizer, reduces ammonia volatilization, delays leaf senescence, and enhances storage nutrition, fruit yield, and fruit quality in peach trees.

## Introduction

Fertilizers are important input materials for the sustainable development of modern orchard production and play a key role in fruit tree yield and quality. China is the world’s largest consumer of fertilizers, accounting for 30% of total global consumption. However, the fertilizer utilization rate is only 30–40% (Zhu, 2000). A low utilization rate of fertilizer is a common problem for chemical fertilizer. Nitrogen loss is particularly serious in chemical fertilizer use. Extensive use of fertilizers leads to a decrease in nitrogen use efficiency, and much of the excess nitrogen fertilizer is lost to the environment (MOA, 2007). This not only results in a decline in fruit quality but also causes soil pollution, water resource pollution, air pollution, and other problems.

Since the beginning of this century, the cultivated area of peach trees in the world has expanded from 12.74×10^5^ ha in 2000 to 17.12×10^5^ ha in 2018 (FAO, 2020). China accounts for 34% of this area, making it the country with the most peach tree cultivation in the world. Peach is the fourth most cultivated fruit tree after apple, citrus, and pear in China. Previous studies showed that the ranges of nitrogen, phosphorus, and potassium fertilization in peach orchards were 495–2,391 kg·ha^−1^ N, 252–1795 kg·ha^−1^ P_2_O_5_, and 323–1,399 kg·ha^−1^ K_2_O, respectively (Kou, 2005; Yuan, 2007; Gao, 2010). However, based on different soil nutrient conditions, Chinese experts recommend the application range of N as 67–320 kg·ha^−1^, P_2_O_5_ 30–160 kg·ha^−1^, and K_2_O 92–346 kg·ha^−1^ for stable peach orchards with a target yield of 40 t·ha^−1^ (Kong et al., 2010; Guo, 2019). Excessive fertilizer application and low fertilizer efficiency lead to a large surplus of nutrients in orchard soil. Surplus nutrients not only are a waste of resources but also cause a series of environmental problems such as greenhouse effects, water eutrophication, soil acidification and biological toxicity. Therefore, scientific management of orchard nutrients is of great significance to conserve fertilizer resources and protect the ecological environment.

Peach trees are perennial woody plants whose growth cycle is divided into two basic processes: life cycle and annual cycle. The annual growth cycle is divided into two periods, growth and dormancy. The growth period is the period from spring germination to the formation of deciduous leaves and includes the three aspects vegetative growth, reproductive growth, and nutrient accumulation. The dormancy stage refers to the stage of deciduous leaf germination. The dormancy period refers to the period from leaf shedding to new bud germination in the second year. Nutrient absorption and utilization in the annual cycle can be divided into four stages, namely, the utilization and storage, an alternate stage involving storage and seasonal nutrients, the seasonal nutrient stage, and the nutrient accumulation and storage stage (Li, 2013). Only when we are familiar with the growth and development characteristics of each period, we can adjust and control the nutrients to match the absorption of nutrients at each stage to reduce the amount of fertilizer to improve the yield and quality. Therefore, the nutrient demand of fruit trees and the nutrient supply and demand in the growing season must be taken into account when formulating fertilization strategies (Weinbaum et al., 1992).

Nutrient loss from fertilizers is associated with soil characteristics, climatic conditions, and the type of fertilizer (Bouwman et al., 1997; Abalos et al., 2014). Among these, only the type of fertilizer is easily controlled. The use of slow- and controlled-release fertilizers (CRFs) is an effective way to solve the problems of resource waste and environmental pollution that would be caused by abuse of chemical fertilizers, especially in China. CRFs, which are coated with polymerized latex, release nutrients in a controlled, delayed manner in synchrony with the sequential needs of plants for nutrients, thereby increasing nutrient utilization efficiency and yield; thus, they provide enhanced nutrient use efficiency along with enhanced yields (Shaviv, 2005). The integration of water and fertilizer can realize the simultaneous supply of nutrients and water and solve the problem of incoordination between water and fertilizer in most orchards in China (Lu, 2013). However, the high price of coated slow/CRF and water-soluble fertilizer with supporting machinery and equipment input makes their use impossible for widespread application in peach orchard production.

Inspired by tea bags, our laboratory has developed a new bag-controlled release fertilizer (BCRF; Peng et al., 2007). It is made by using a paper-plastic composite material to wrap various kinds of fertilizers such as nitrogen, phosphorus, and potassium. Fertilizers release nutrients to the environment through microholes in the bag. In recent years, our research found that the nutrient release rate of BCRF was basically consistent with the fertilizer requirement of fruit trees, which could promote the occurrence of fine roots, significantly improve the nitrogen utilization rate, and improve the comprehensive quality of fruit. Our laboratory has obtained the initial results for applying BCRF, a new fertilizer with an environmentally safe material and a high utilization rate, for fruit tree production. At present, there are many studies on improving the nitrogen utilization rate of BCRF (Peng et al., 2006; Zhang et al., 2008; Jiang et al., 2015), while few studies on reducing nitrogen loss (such as ammonia volatilization) have been conducted. Du et al. (2011) and Wang et al. (2013) found that polymer-coated fertilizer (PCF) can reduce NH_3_ volatilization and N_2_O emissions, because the nutrient release mechanisms between BCRF and PCF are similar to a large extent.

BCRF may play a positive role in regulating peach root growth, root turnover, leaf senescence, nitrogen absorption and utilization efficiency, and fruit yield and quality. Therefore, in this study, we evaluated the effects of BCRF on ammonia volatilization, growth and senescence, and storage nutrient content of peach trees. Furthermore, we examined the impact of the application of BCRF for 5 continuous years on root growth, nitrogen utilization rate, and fruit yield and quality to provide a reference for scientific fertilization in peach orchards.

## Materials and Methods

### Preparation of Bag and Fertilizer Composition

**Figure 1:**
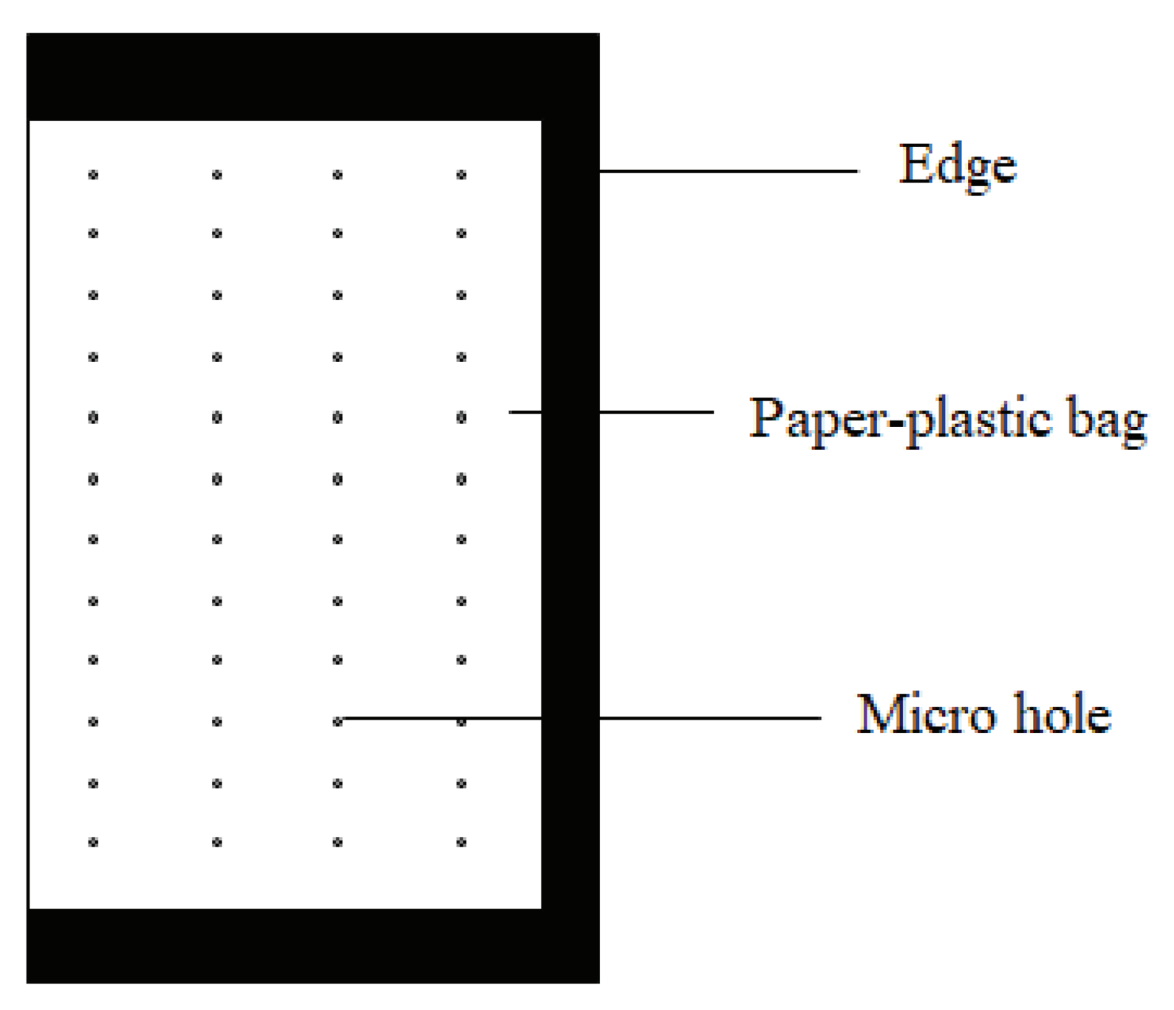
Bag of bag-controlled release fertilizer (BCRF).

The bag was made of a paper-plastic composite material as described in our patented product (Figure 1; Peng et al., 2007). Rows of micro holes were uniformly distributed on both sides of the bag. The diameter of the microholes was 0.2 mm, and the distance between micro holes was 0.5 cm. The fertilizer in the bags consisted of urea, diammonium hydrogen phosphate, and potassium sulfate in a ratio of 41:14:40 (N:P_2_O_5_:K_2_O). After blending, the composite fertilizer was packaged into the bags by a granular packager with paper-plastic composite material. The bags were 9 cm wide and 15 cm long (20 g/bag, BCRF I and 95 g/bag, BCRF II).

### Plant Materials and Experimental Treatments

We conducted two experiments: one is pot experiment and another one is field experiment.

#### Pot Experiment

To study the effects of the application of BCRF on plant growth and senescence, storage nutrition, and NH_3_ volatilization, 2-year-old “Jingqing14”/wild peach (*Amygdalus persica* Linn.) seedlings were used as materials, and a pot experiment was carried out. On March 25, peach seedlings were planted in a pot with a diameter of 30 cm and a height of 45 cm. Three experimental treatments were used: control (no fertilizer application), BCRF I (20 g/bag, paper-plastic bag with the mixed fertilizer as described above), and FSA I (20 g/bag, common nylon bag with 0.178 mm hole size instead of the paper-plastic bag). The fertilizers BCRF I and FSA I were applied to two bags per tree on both sides of the trunk. Three plots were set up for each treatment, and each plot contained seven trees. Irrigation was carried out when the field capacity was less than 50%, and the irrigation amount was 2.5 L in each pot each time. A single factor random block design was employed for the experiment, and each treatment was performed in triplicate.

#### Field Experiment

To study the effects of BCRF application on the soil nutrient status, nitrogen absorption and distribution, root growth, and yield and quality in the peach orchard, the late-ripening peach “Ruipan 21”/wild peach [*Prunus persica* (*Carr.*) Franch.] was used, and a field experiment was conducted from 2012 to 2016 with two fertilization modes: BCRF II (95 g/bag, paper-plastic bag with the mixed fertilizer as described above) and equal amounts of FSA II. Three plots were set up for each treatment, and each plot contained six trees. The fertilizers were applied by the radiating ditch method, that is, a radiating ditch was dug outward 30 cm from a trunk, with a width of 15–20 cm, a depth of 20–30 cm, and a length of 20–30 cm. The radiating ditch position was changed every year. The fertilizer application is shown in Table 1. To determine nitrogen absorption and utilization, in 2016, 0.5 g of ^15^N urea was used for each bag of BCRF treatment instead of 0.5 g of ordinary urea, and 5 g of ^15^N urea was used to replace 5 g of ordinary urea in the FSA I treatments for mixed application. A single factor random block design was employed for the experiment, and each treatment was performed in triplicate.

**Table 1:** Fertilizing dates and amounts.

Treatment	January 4, 2012	January 3, 2013	January 3, 2014	January 3, 2015	January 3, 2016
	Number of radial ditches × Amount of bag-controlled release fertilizer (bag/ditch)				
BCRF II	2 × 1	4 × 1	4 × 2	5 × 2	5 × 2
FSA II	2 × 1	4 × 1	4 × 2	5 × 2	5 × 2

### Measurement of Plant Growth and Senescence Parameters

After the new shoot stopped growing (September 30), the growth indexes, such as the total length of the new shoot, dry stem, and branch stem, were measured. The net photosynthetic rate of peach leaves was measured with a CIRAS-3 portable photosynthetic measurement system (PP Systems, United Kingdom), and the chlorophyll content was measured as the SPAD value by using an SPAD-502 chlorophyll meter (Tuopu Yunnong Technology). Root activity was measured by the triphenyltetrazolium chloride (TTC) method (Zhao et al., 2002).

Enzyme extracts from 0.5 g samples for the determination of superoxide dismutase (SOD), catalase (CAT), and peroxidase (POD) activities were prepared with chilled phosphate buffer. The homogenate was centrifuged at 10,000 *g* for 20 min at 4°C. The supernatant was referred to as the enzyme extract. The SOD activity was determined by the nitrogen blue tetrazole (NBT) method and calculated according to the method of Wang et al. (2009). The CAT activity was measured as previously described (Kar and Mishra, 1976). One unit of enzyme activity (U) was defined as the amount required to change the OD_240_ by 0.1 within 1 min. POD activity was determined by the guaiacol method (Omran, 1980), and the enzyme activity unit (U) was determined as the amount of enzyme required to reduce the OD_470_ by 0.1 per minute. Malondialdehyde (MDA) content was determined by the thiobarbituric acid (TBA) method (Zhao et al., 2002).

### Collection and Measurement of NH_3_ Gas

NH_3_ gas was collected as previously described (Zhu et al., 1985) with some improvements. A split plastic bucket with an inner diameter of 30 cm and a height of 20 cm was inverted on the soil surface centered on the fertilization site. There was a plastic box containing 200 ml of 2% boric acid and indicator in the bucket, and boric acid was changed over time. NH_3_ samples were collected every 2 days in the first 30 days, followed by once every 10 days until ammonia volatilization was not detected. The NH_3_ absorbed in boric acid was titrated by 0.005 mol·L^−1^ H_2_SO_4_.

NH_3_ volatilization rate (mg/day) = the average amount of NH_3_ volatilization measured per time (m_i_)/continuous capture period length in every sampling (day).

Cumulative amount of NH_3_ volatilization (mg) = m_1_ + m_2_ + m_3_ + ··· + m_i_.

### Determination of Soluble Sugar and Starch

The soluble sugar and starch contents were measured as previously described (Wang et al., 2012). Soluble sugar was extracted from a 1 g sample that had been cut into small pieces, put into a tube and then extracted twice at 100°C for 30 min. The filtered solution was transferred into a 25 ml volumetric flask, and the soluble sugar content was determined by the anthrone colorimetric method. The residue after extraction of soluble sugar was transferred into a 10 ml graduated test tube that was capped with a stopper. The residue was digested with 0.92 mol·L^−1^ perchloric acid at 100°C in 10 ml of water for 15 min to convert starch to glucose. Glucose was used for the determination of starch content by the anthrone colorimetric method.

### Determination of Soluble Protein and Free Amino Acid

Soluble protein was extracted from a 1 g sample with extraction buffer consisting of 25 mmol·L^−1^ potassium phosphate buffer (pH 7.5), 5 mmol·L^−1^ EDTA-Na_2_, and 5 mmol·L^−1^ cysteine. The sample (1 g) was ground in 3 ml of extraction buffer consisting of 25 mmol·L^−1^ potassium phosphate buffer (pH 7.5), 5 mmol·L^−1^ EDTA-Na_2_, and 5 mmol·L^−1^ cysteine. The suspension was transferred to centrifuge tubes and clarified by centrifugation for 15 min at 12,000 × *g* at 4°C. The supernatant was used for the measurement of soluble protein content by the Coomassie brilliant blue method using bovine serum albumin as the standard protein.

Free amino acids were extracted from 1 g of each sample that had been placed in a tube with 10 ml of water at 100°C and then extracted twice with the same volume of water at 100°C. The filtered solution was transferred into a 25 ml volumetric flask, and the free amino acid content was determined by the ninhydrin colorimetry method using leucine as the standard amino acid.

### Determination of New Root Growth Status

**Figure 2:**
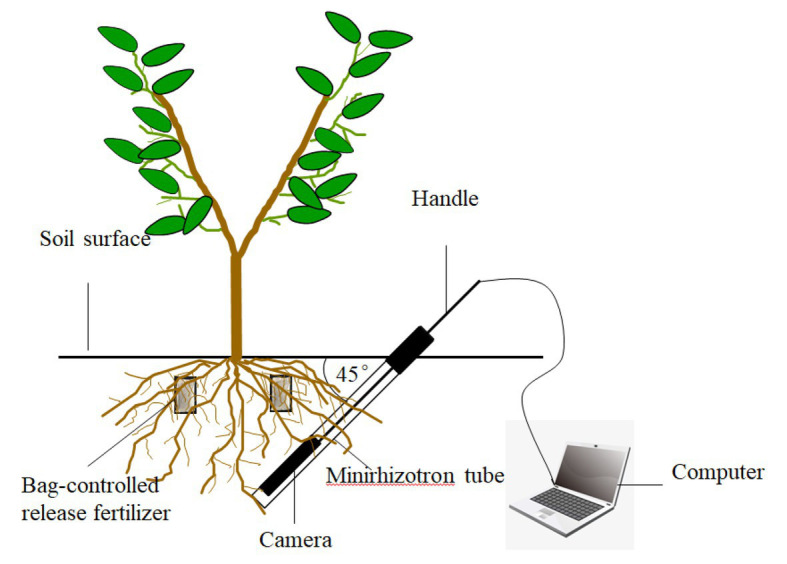
Installation of minirhizotron tube.

As shown in Figure 2, new root growth was observed using the root *in situ* observation system PMT-Root Scanner-R (Analysis, Beijing, China). On April 1, 2016, six peach trees were randomly selected from each treatment; root observation tubes were buried 0.6 m from the trunk at a 45° angle from the ground, and the buried depth was 45 cm. Images of the 10–30 cm soil layer were collected every 15 days from April 15 to December 15, and the image collection area was 21.56 cm × 19.56 cm.

Root biomass was analyzed using the software of the system. White and brown roots were defined as living roots. Black or shriveled roots and roots that disappeared during the two observation periods were defined as dead roots.

Annual turnover rate of fine roots = Annual dead fine root biomass/Annual maximum fine root standing crop.

### Analysis of Root Horizontal Distribution Characteristics

Three plants were selected for each treatment, and the root samples were collected by the sectional digging method on November 2016. With the trunk as the center and every 20 cm in the horizontal direction between the rows, square holes (50 cm × 20 cm × 40 cm) were dug until no roots appeared. The root samples were collected, labeled at every segment, and then analyzed by a WinRHIZO automatic root system scanner.

### Determination of Nitrogen Absorption and Utilization

In the fruit maturity period (on September 20, 2016), samples were collected from the peach trees that were labeled with ^15^N urea for analysis. The tree was divided into six parts: main trunk, lateral branches, leaves, thick roots (diameter > 2 mm), fine roots (diameter < 2 mm), and fruits. Then, every sample was rinsed successively with clear water, detergent, clear water, 1% hydrochloric acid, and three times with deionized water. Immediately, the samples were dehydrated at 105°C for 30 min and then dried at 80°C to constant weight. After that, the samples were ground and crushed through a 0.147 mm sieve and mixed for determination. The total nitrogen content was measured using the Kjeldahl method (Bao, 2000), and the ^15^N abundance was determined by a MAT-251 mass spectrometer (College of Resource and Environment, Shandong Agricultural University).

Nitrogen utilization rate equations:

Ndff=N 15 abundanceinplantsample−N 15 naturalabundance/N 15 abundanceinfertilize−N 15 naturalabundance×100%.

$$\begin{array}{l} \mathrm{Nitrogen utilization rate}=\left[\mathrm{Ndff}\times \mathrm{total nitrogen content of organ}\;\left(g\right)\right]/\\ \mathrm{amount of fertilizer applied}\;\left(g\right)\times 100\%. \end{array}$$

### Determination of Fruit Yield and Quality

The fruit yield and quality were counted and analyzed every year after the trees had borne fruit. The fruit were collected from the middle to the top of the peach trees for measurement of fruit quality. Soluble solid content was measured using a TD-65 digital refractometer (Zhejiang Top Instrument). Titratable acid content was measured using the acid-base titration method. Vitamin C content was measured using the iodine titration method.

### Statistical Analysis

Each measurement was repeated three times, and the mean values of each parameter were calculated. The statistical analysis was performed with Microsoft Office Excel 2010 software. Statistical analyses of the data were performed using DPS statistical package, and the difference were statistically compared by employing the Duncan test with a significance level of *p* < 0.05.

## Results

### BCRF Can Promote the Shoot Growth of Peaches

Compared with the FSA I treatment, the BCRF I treatment significantly promoted the growth and development of young peach trees. BCRF treatment increased trunk straight stem growth, primary lateral branch lengths, and primary lateral branch thickness by 26.22, 35.11, and 20.85%, respectively. The number of secondary branches was reduced in the BCRF I treatment (Table 2).

**Table 2:** Aboveground biomass of peach trees under different treatments.

Treatment	Trunk straight stem growth (mm)	Primary lateral branch length (cm)	Primary lateral branch thickness (mm)	Secondary lateral branch number
Control	3.06 ± 0.08^b^	212.09 ± 7.56^c^	6.04 ± 0.15^b^	4.15 ± 0.35^c^
FSA I	3.28 ± 0.07^b^	289.02 ± 9.98^b^	6.33 ± 0.14^b^	8.89 ± 0.42^a^
BCRF I	4.14 ± 0.06^a^	390.50 ± 15.07^a^	7.65 ± 0.20^a^	5.22 ± 0.42^b^

Different letters indicate statistically significant differences (*p* < 0.05).

### BCRF Can Delay Leaf and Root Senescence

**Figure 3:**
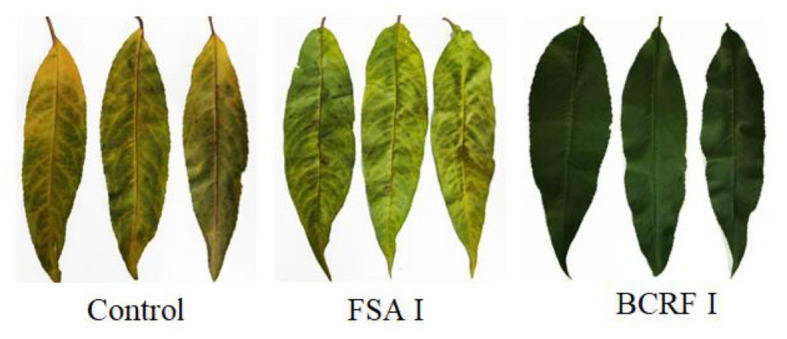
Phenotype of peach leaves under different fertilization modes.

**Figure 4:**
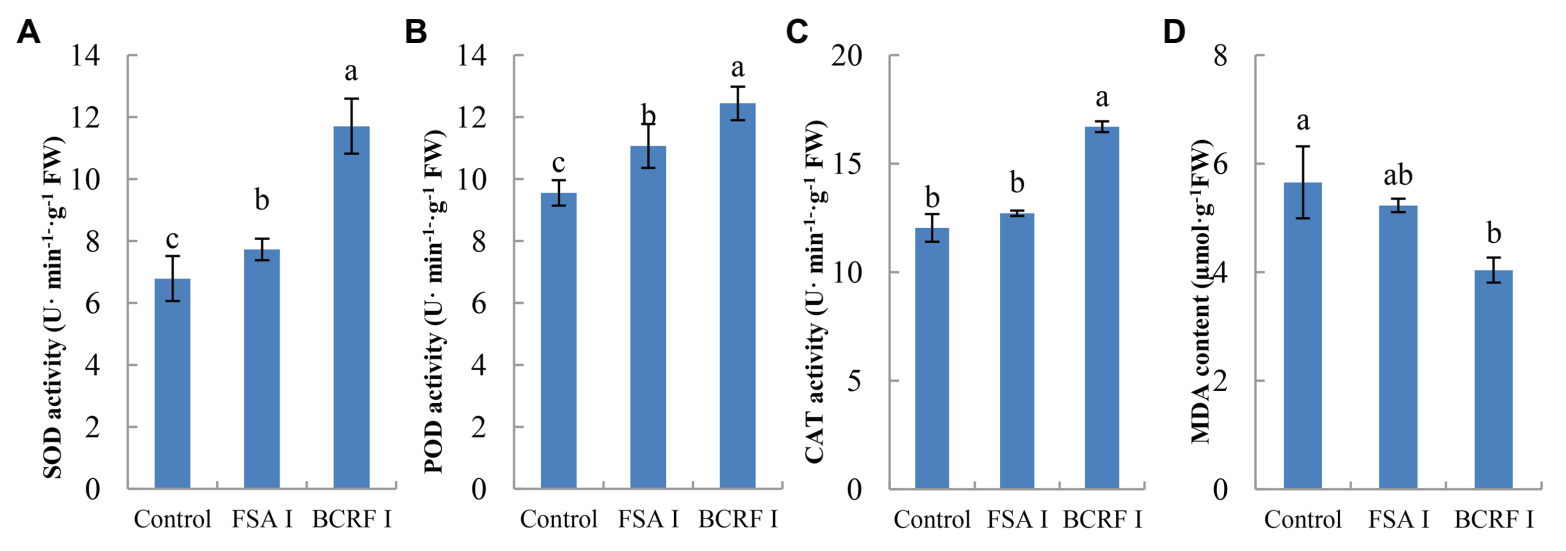
Effects of bag-controlled release fertilizer on superoxide dismutase (SOD; **A**), peroxidase (POD; **B**), and catalase (CAT; **C**) activities and malondialdehyde (MDA) content **(D)** in peach tree leaves. The vertical bars indicate the standard deviation of three replications. Different letters indicate statistically significant differences (*p* < 0.05).

Leaf senescence is an important feature in late stages of plant development. When the leaves of plants are aging, photosynthesis decreases, which greatly affects the production by plants. The most significant morphological change in leaf senescence is the change in leaf color. From Figure 3, we can see that BCRF I delayed leaf senescence. On October 1, we measured the net photosynthetic rate and chlorophyll content of peach leaves and found that those of trees treated with BCRF I were significantly increased (Table 3) compared with those of trees receiving the control treatment. As shown in Figure 4, compared with FSA I treatment, BCRF I treatment led to a significant increase in antioxidant enzyme (SOD, POD, and CAT) activities and a decrease in MDA content consistent with the leaf phenotype.

**Table 3:** Leaf net photosynthetic rates and chlorophyll content under different treatments.

Treatment	Pn (μmol·m^2^·s^−1^)	Chlorophyll content (SPAD value)
Control	7.08 ± 0.15^b^	23.93 ± 0.13^b^
FSA I	7.28 ± 0.17^b^	24.50 ± 0.18^b^
BCRF I	11.68 ± 0.13^a^	33.53 ± 0.15^a^

Different letters indicate statistically significant differences (*p* < 0.05).

**Figure 5:**
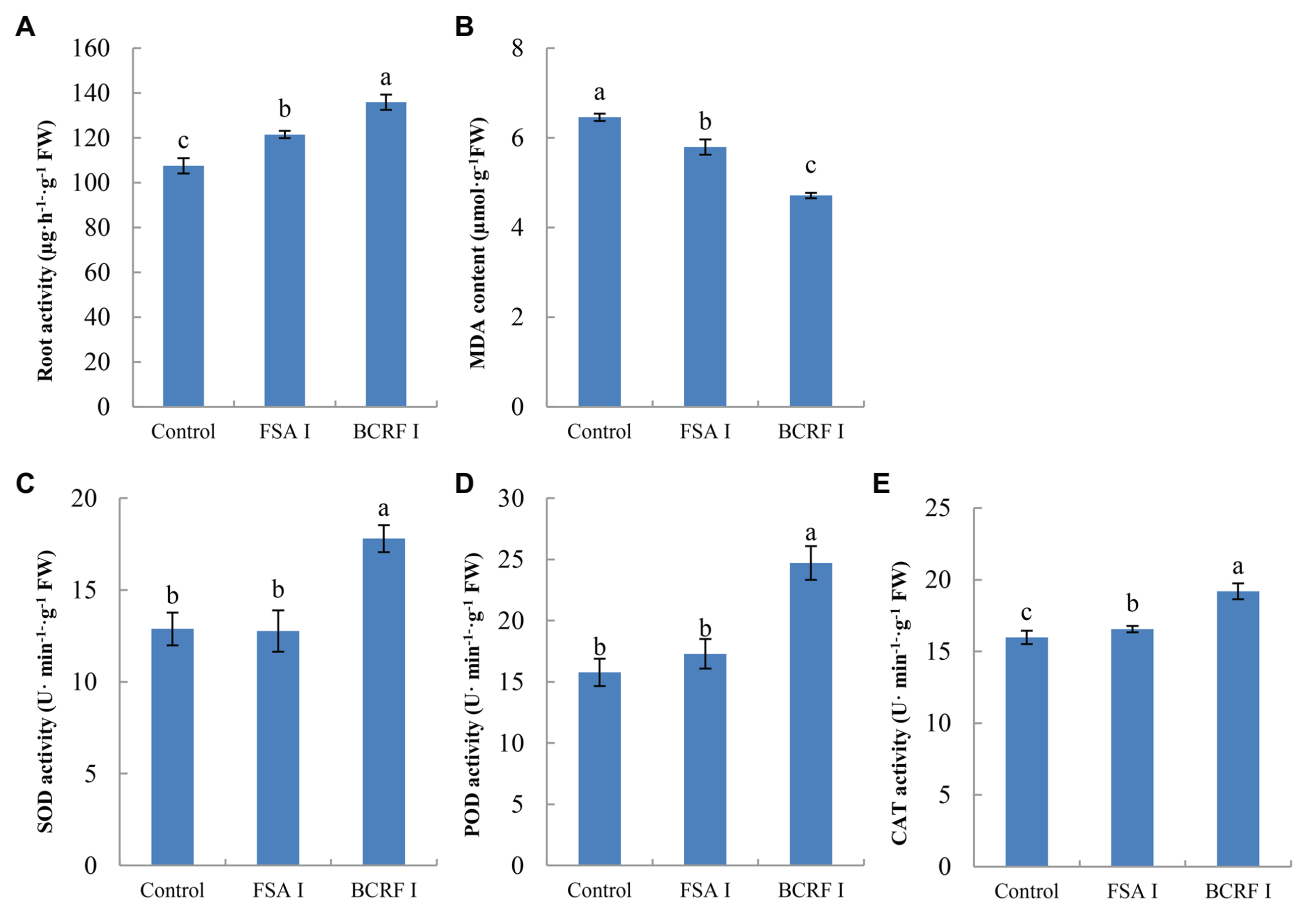
Effects of bag-controlled release fertilizer on root activity **(A)**, MDA content **(B)** and SOD **(C)**, POD **(D)**, and CAT **(E)** activities. The vertical bars indicate the standard deviation of three replications. Different letters indicate statistically significant differences (*p* < 0.05).

The root is an active absorbing organ, and root activity directly affects the absorption of nutrients and growth aboveground. We analyzed the root activity and found that it was 17.53% higher in the BCRF I treatment than in the FSA I treatment. The SOD, POD, and CAT activities were higher in the BCRF I treatment than in the FSA I treatment, and the MDA content was reduced in the BCRF I treatment (Figure 5). Thus, the BCRF I treatment delayed root senescence.

### BCRF Can Reduce NH_3_ Volatilization From Soil

**Figure 6:**
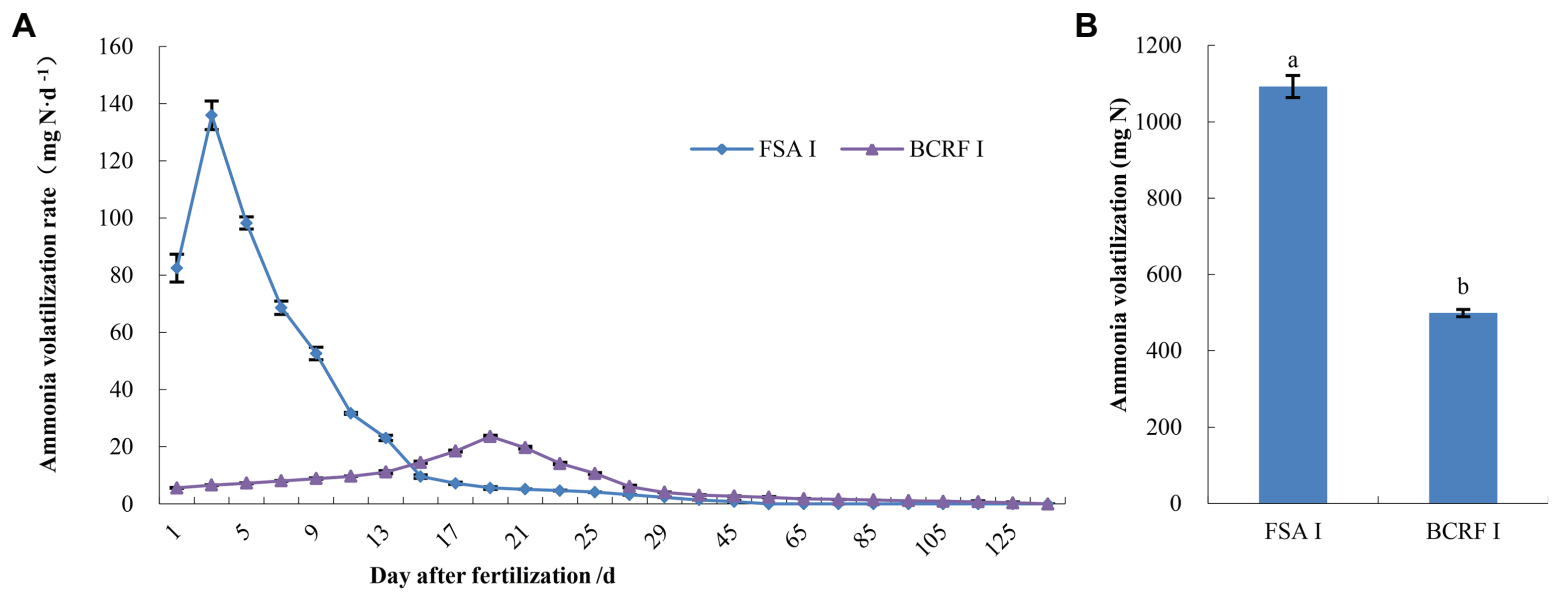
Effects of BCRF on NH_3_ volatilization. **(A)** Ammonia volatilization rate and **(B)** cumulative amount of ammonia volatilization. Different lowercase letters indicate significant differences according to t-tests (*p* < 0.05).

Ammonia volatilization is an important path of nitrogen loss in peach orchards. Compared with FSA I, BCRF I decreased ammonia volatilization (Figure 6). The ammonia volatilization rates of BCRF I and FSA I soils were significantly different. Volatilization peaks were achieved on day 3 for FSA I after fertilizer application, and no volatilization was detected on day 50. Volatilization reached its peak on day 19 for BCRF I. Subsequently, volatilization gradually decreased, and no volatilization was detected on day 120 (Figure 6A). As shown in Figure 6B, the cumulative amount of ammonia volatilization for the FSA I treatment was 1092.30 mg, which was 2.19 times that for BCRF I treatment. The ammonia volatilization loss under the FAS I treatment accounted for 13.76% of the applied nitrogen, while loss under BCRF I accounted for only 6.28%. BCRF significantly reduced ammonia volatilization loss.

### BCRF Affects the Storage Carbon and Nitrogen Nutrition in Peaches

**Figure 7:**
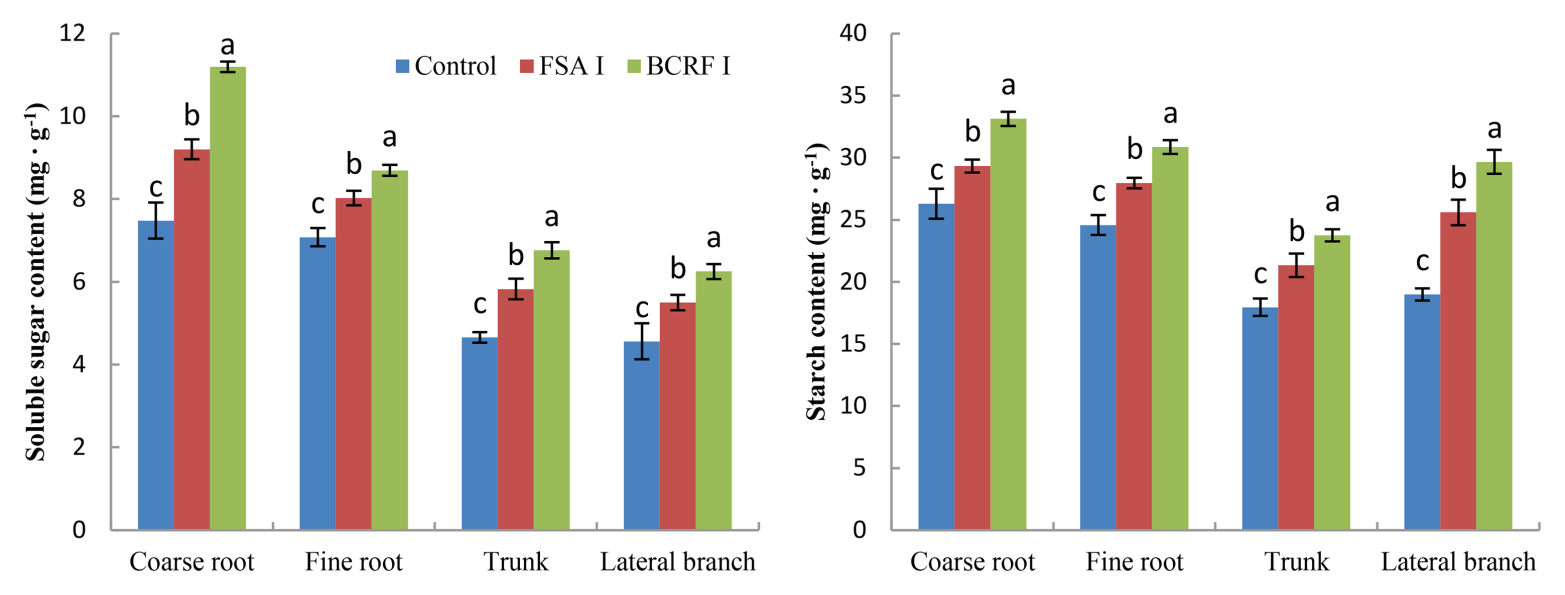
Effects of BCRF on soluble sugar content and starch content in different organs. The vertical bars indicate the standard deviation of three replications. Different letters indicate statistically significant differences (*p* < 0.05).

**Figure 8:**
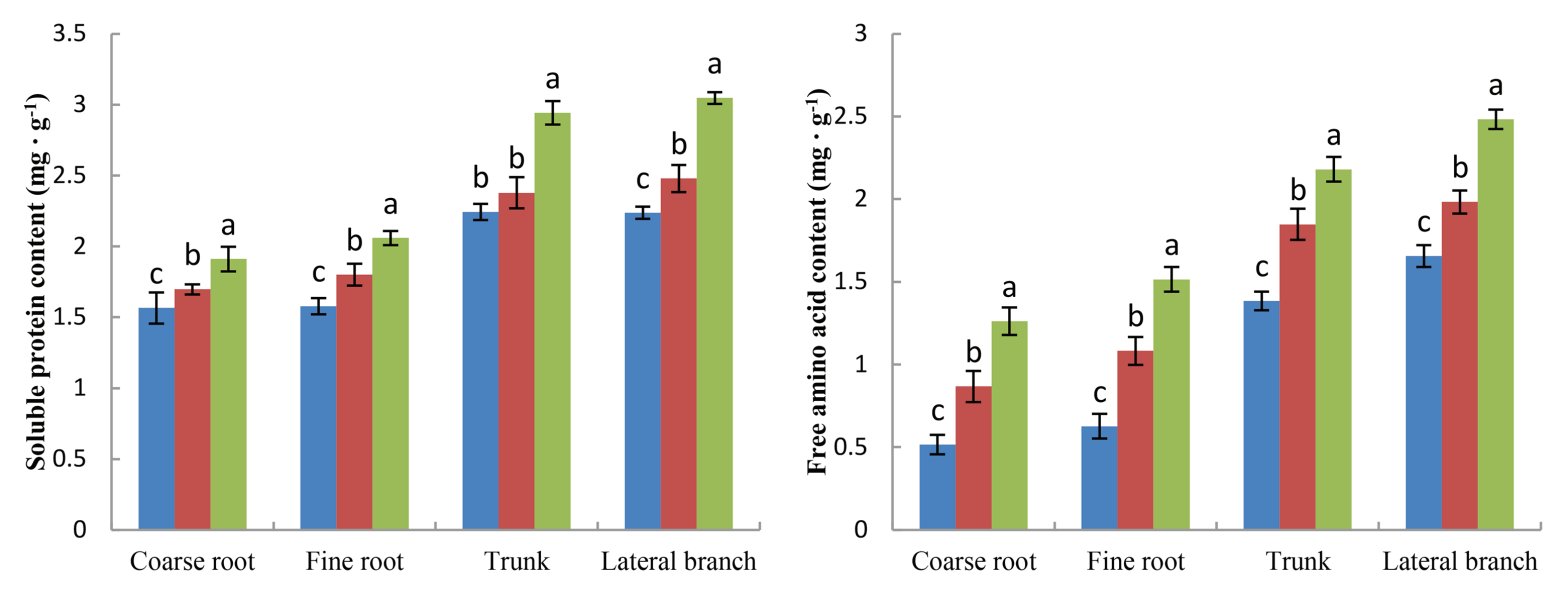
Effects of BCRF on soluble protein content and free amino acid content in different organs. The vertical bars indicate the standard deviation of three replications. Different letters indicate statistically significant differences (*p* < 0.05).

The term storage nutrients refer to substances that are not used immediately but are mainly stored in the roots, trunks, and branches. Storage nutrients, including storage carbon and storage nitrogen, play an important role in the safe overwintering and growth of fruit trees in the next year. BCRF I significantly increased the storage nutrition content of peach trees (Figures 7, 8). We analyzed the storage carbon nutrients in peach trees and found that 56.7% were stored in roots and 43.3% in trunk and branches. The soluble sugar content and starch content in peach trees treated with BCRF I were 15.2 and 12.6% higher than those in trees treated with FSA I, respectively. Storage nitrogen nutrients mainly include soluble protein and free amino acids. As shown in Figure 8, 38% of the storage nitrogen nutrients in peach trees were stored in roots, and 62.0% were stored in trunk and branches. The content of storage soluble protein and free amino acids in peach trees for the BCRF I treatment increased by 19.2 and 28.7%, respectively, compared with those for the FSA I treatment.

### BCRF Can Promote New Root Production and Enhance the Root Turnover Rate

**Figure 9:**
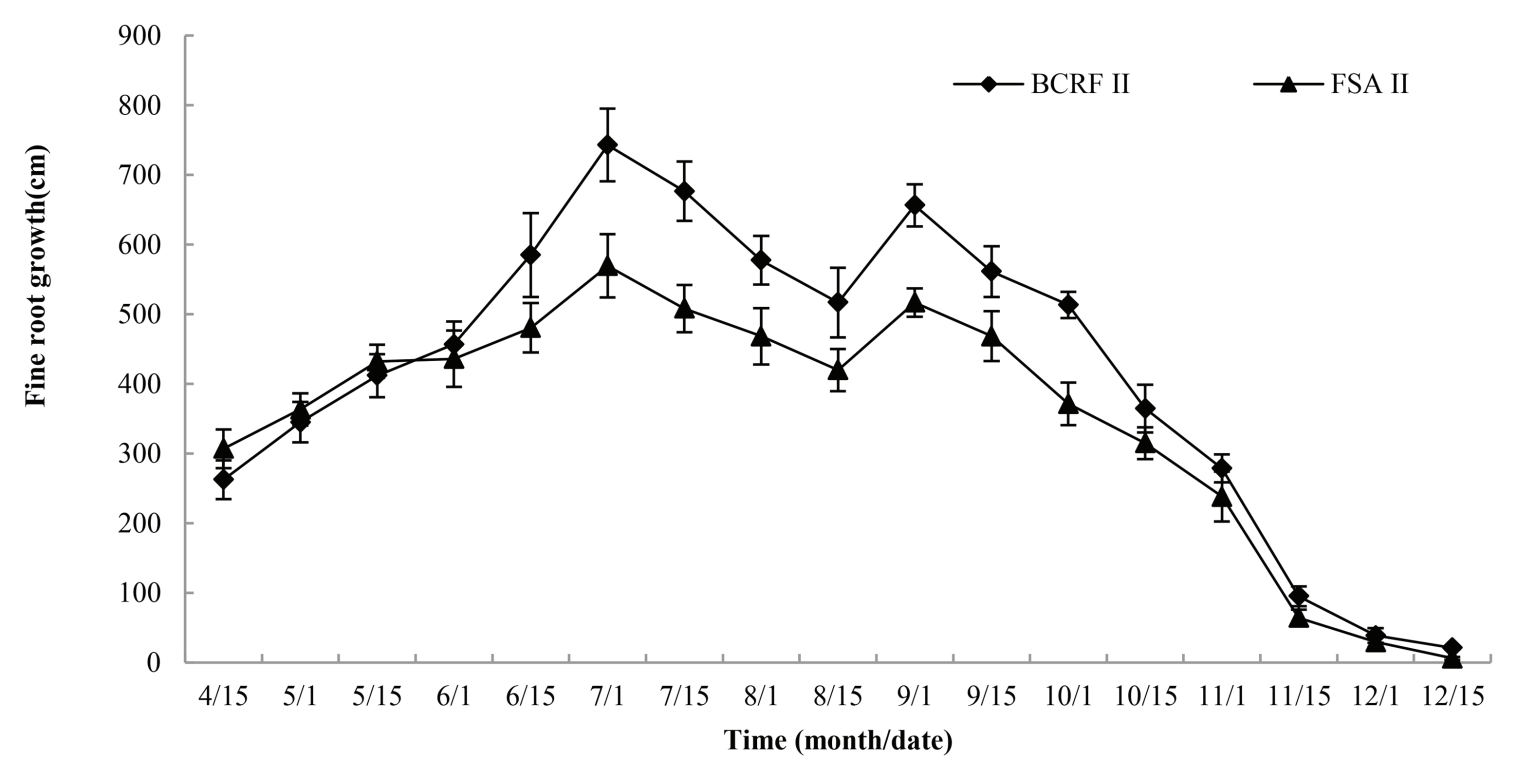
Growth dynamics of peach fine roots under different fertilization modes. The vertical bars indicate the standard deviation of three replications.

**Figure 10:**
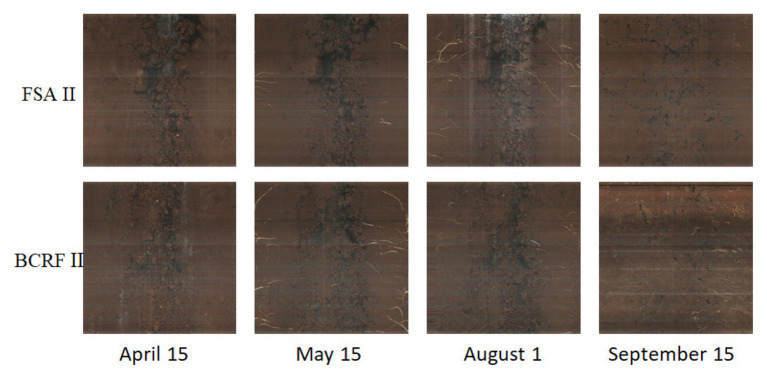
Root scanning pictures of peach trees under different fertilization modes.

To study the influence of BCRF on root occurrence dynamics, the Root Scanner-R system was used to collect root pictures every 15 days from April 15 to December 15. The roots had two growth peaks in a year, in July and September, and then the root growth slowed down in November and basically stopped in mid-December. Before June, the occurrence of new roots under the FSA II treatment was faster than that under the BCRF II treatment. However, after June, new roots under the BCRF II treatment formed significantly faster than those under the FSA II treatment (Figure 9). As shown in Figure 10, on September 15, FSA II treated peach trees had fewer new roots, while BCRF II treated peach trees still had more white new roots. Meanwhile, it was more intuitive to show that BCRF could delay root senescence.

We also analyzed the annual fine root production biomass, dead fine root biomass, and maximum fine root standing crop. The results showed that the annual fine root production biomass and maximum fine root standing crop under the BCRF II treatment were significantly higher than those under the FSA II treatment, and there was no significant difference in the annual dead fine root biomass. Moreover, BCRF decreased the turnover rate of fine roots (expressed in terms of annual dead fine root biomass/maximum fine root standing crop; Table 4). This indicated that the growth of fine roots was relatively active and that BCRF slowed the death rate of the roots and improved the root life span.

**Table 4:** Fine root turnover rate of peach trees.

Year	Treatment	Annual fine root production biomass (cm)	Annual dead fine root biomass (cm)	Annual maximum fine root standing crop (cm)	Turnover rate
2013	BCRF II	7116.49 ± 152.14^a^	1327.49 ± 69.83^a^	10453.93 ± 394.82^a^	0.127 ± 0.010^b^
	FSA II	5990.41 ± 138.28^b^	1384.31 ± 77.49^a^	9627.01 ± 261.98^b^	0.144 ± 0.007^a^
2014	BCRF II	9916.64 ± 190.88^a^	2079.01 ± 109.33^a^	13208.51 ± 285.82^a^	0.158 ± 0.012^b^
	FSA II	8162.54 ± 267.28^b^	2138.95 ± 163.12^a^	11930.17 ± 120.54^b^	0.179 ± 0.011^a^

Different letters indicate statistically significant differences (*p* < 0.05).

### BCRF Can Increase the Proportion of Fine Roots

**Figure 11:**
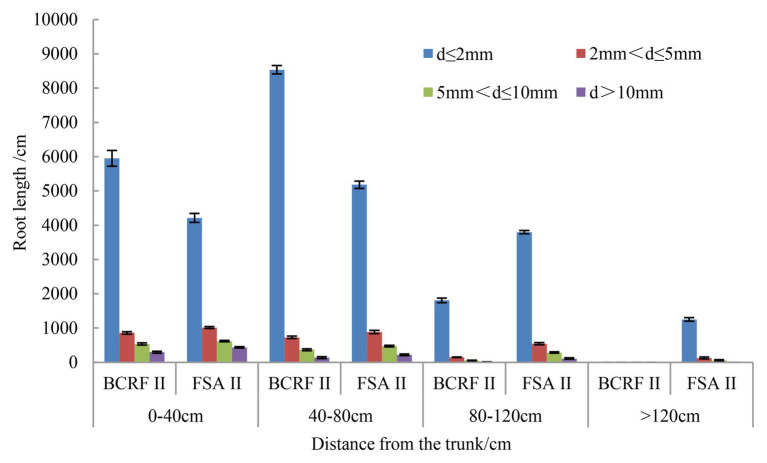
Root horizontal distributions under different fertilization modes. The vertical bars indicate the standard deviation of three replications.

The distribution of peach roots reached more than 120 cm in the horizontal direction under the FSA II treatment and less than 120 cm under the BCRF II treatment. In addition, 88.91% of fine roots (day ≤ 2 mm) were distributed from 0 to 80 cm under the BCRF II treatment, while in the FSA II treatment, 71.61% of the fine roots were distributed in 0–80 cm (Figure 11). We also analyzed the proportions of fine roots and found that fine roots accounted for 83.95 and 75.19% of the total root length under the BCRF II and FSA II treatments, respectively. It was further proven that BCRF could promote the occurrence of fine roots.

### BCRF Can Increase the Nitrogen Utilization Rate in Peach Trees

The total N content, Ndff (N derived from fertilizer), and N utilization rate are shown in Table 5. The Ndff values in different organs for BCRF II were significantly higher than those for FSA II. BCRF II plants showed a higher total N (223.83 g) and N utilization rate (34.46%) than FSA II plants. BCRF II significantly improved the N utilization rate in the aboveground parts of the peaches, and those in the fruit, leaf, main trunk, and lateral branch were 1.58, 1.22, 1.51, and 1.48 times those for the FSA II treatment, respectively. FSA II led to a drastic increase in available nutrient concentration within a short period of time. High concentrations of available nutrients are not rapidly absorbed by plants, and most are lost by nitrification, denitrification, ammonia volatilization, or entry into the groundwater system. However, BCRF II results in slow nutrient loss and decreases the effects of changes in soil moisture on absorption by peach trees, resulting in a significantly higher N utilization rate in peach trees.

**Table 5:** Effects of different fertilization modes on the nitrogen utilization rate.

Organ	Treatment	Dry mass (g/plant)	Total N (g)	Ndff (%)	N utilization rate (%)
Leaf	BCRF II	1228.66 ± 38.91^b^	33.83 ± 1.43^a^	1.20 ± 0.03	8.15 ± 0.26^a^
	FSA II	1318.84 ± 40.44^a^	32.09 ± 1.04^ab^	1.04 ± 0.01	6.69 ± 0.25^b^
Main trunk	BCRF II	13684.38 ± 613.27^a^	61.09 ± 2.41^a^	0.32 ± 0.01	3.97 ± 0.31^a^
	FSA II	12314.56 ± 297.94^b^	50.88 ± 2.18^b^	0.26 ± 0.03	2.63 ± 0.27^b^
Lateral branch	BCRF II	9146.97 ± 185.12^b^	71.96 ± 0.92^a^	0.28 ± 0.01	4.01 ± 0.18^a^
	FSA II	10576.47 ± 362.47^a^	73.36 ± 4.00^a^	0.19 ± 0.03	2.71 ± 0.38^b^
Thick root	BCRF II	1959.02 ± 26.21^b^	13.91 ± 0.68^b^	1.26 ± 0.03	3.51 ± 0.19^a^
	FSA II	2210.66 ± 28.70^a^	16.72 ± 0.57^a^	0.97 ± 0.04	3.25 ± 0.10^a^
Fine root	BCRF II	611.28 ± 11.78^a^	9.62 ± 0.52^a^	1.51 ± 0.03	2.90 ± 0.22^a^
	FSA II	592.35 ± 17.86^b^	8.02 ± 0.41^b^	1.24 ± 0.03	1.99 ± 0.13^b^
Fruit	BCRF II	6001.25 ± 135.04^a^	33.41 ± 1.07^a^	1.79 ± 0.02	11.93 ± 0.19^a^
	FSA II	4500.61 ± 63.19^b^	25.05 ± 0.48^b^	1.51 ± 0.03	7.57 ± 0.19^b^
Plant	BCRF II	32631.56 ± 630.50^a^	223.83 ± 1.78^a^		34.46 ± 0.42^a^
	FSA II	31513.50 ± 650.61^b^	206.12 ± 4.42^b^		24.84 ± 0.90^b^

Different letters indicate statistically significant differences (*p* < 0.05).

### BCRF Can Improve the Yield and Quality of Peaches

**Figure 12:**
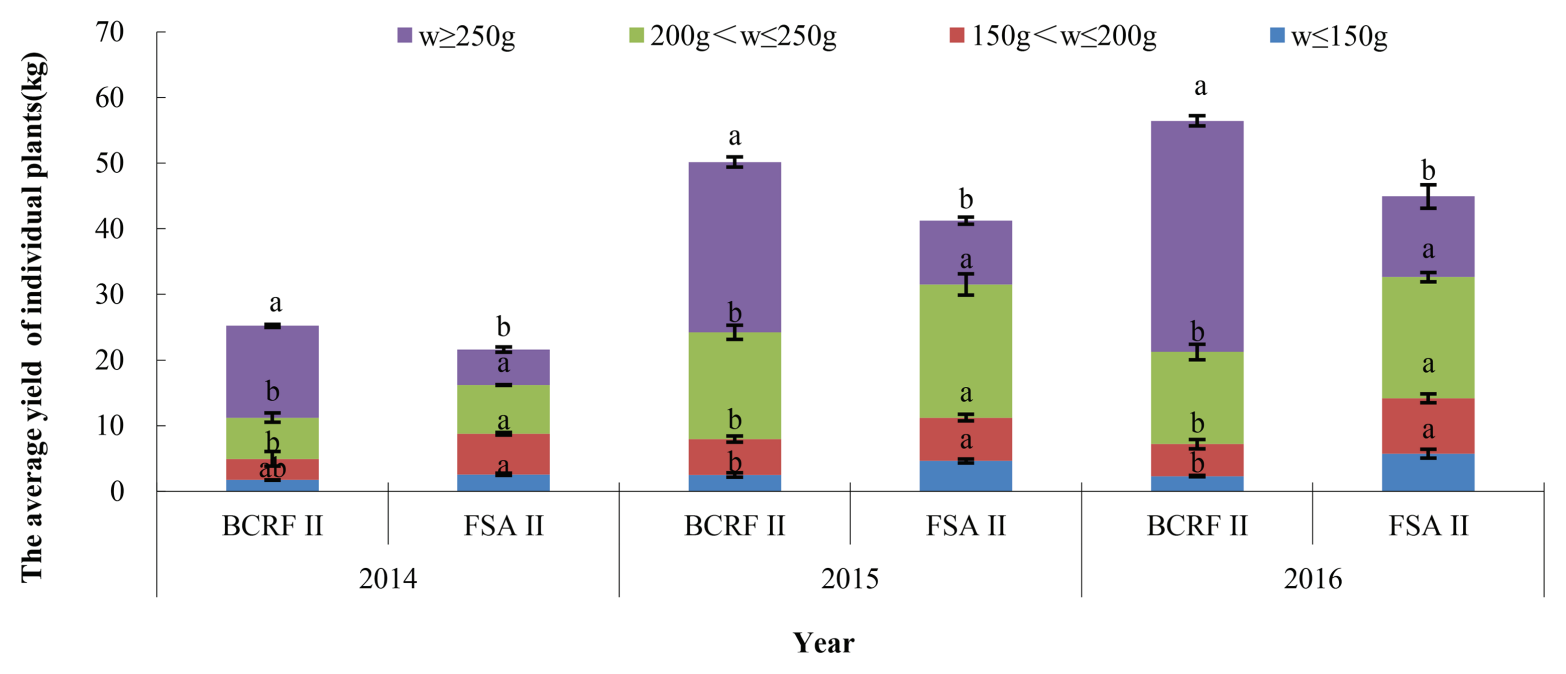
The yield of individual plants under different fertilization modes. The vertical bars indicate the standard deviation of three replications. Different letters indicate statistically significant differences (*p* < 0.05).

Based on the results of 3 consecutive years, compared with the FSA II treatment, the BCRF II treatment increased the yield of individual plants by 16.85, 21.58, and 25.63%, respectively, from 2014 to 2016. According to the weight of each fruit, they were divided into four grades: oversized fruit (w ≥ 250 g), large fruit (200 g < w ≤ 250 g), middle fruit (150 g < w ≤ 200 g), and small fruit (w ≤ 150 g). Oversized and large fruits accounted for more than 80% of the total fruit under BCRF II treatment, while FSA II treatment produced only 59–73% oversized and large fruits (Figure 12). The yields of individual plants and the weights of single fruit were higher in the BCRF II treatment than in the FSA II treatment. From Table 6, we can see that the fruit quality was slightly improved under the BCRF treatment from 2014 to 2016. However, the mean difference in the different fruit quality indexes between the BCRF II and FSA II treatments was not significant.

**Table 6:** The peach fruit quality under different fertilization modes.

Year	Treatment	Soluble solid content (%)	Titratable acid (%)	Soluble solid/acid	Vc (mg∙kg^−1^)
2014	BCRF II	12.72^a^	0.193^a^	65.91^a^	534.02^a^
	FSA II	12.57^a^	0.198^a^	63.49^a^	535.39^a^
2015	BCRF II	12.51^a^	0.186^a^	67.26^a^	525.9^a^
	FSA II	12.46^a^	0.186^a^	66.99^a^	529.73^a^
2016	BCRF II	12.29^a^	0.189^a^	65.03^a^	515.12^a^
	FSA II	12.13^a^	0.186^a^	65.22^a^	512.27^a^

Different letters indicate statistically significant differences (*p* < 0.05).

### Popularization and Application of BCRF in the Demonstration Peach Orchards of Ningxia, Yunnan, and Shandong Provinces

We cooperated with the Fruit Agricultural Science and Technology Co., Ltd., Beijing, and have applied BCRF II for 3–4 years in demonstration peach orchards in Ningxia, Yunnan, and Shandong provinces. The amount of fertilizer input was 188 kg·ha^−1^ in the first year, 376 kg·ha^−1^ in the second and third years, and 500 kg·ha^−1^ in the fourth year. We found that the application of BCRF in peach-producing areas in the three provinces could reduce fertilizer consumption, and the average peach yield did not decrease compared with those of common orchards (Xiao et al., 2019). We will continue to demonstrate the successful application of BCRF in peach orchards in these three provinces (Table 7).

**Table 7:** BCRF application amount and peach fruit yield in three provinces.

Area	Orchard area (ha)	Year	Fertilizer input (kg·ha^−1^)	Yield (kg·ha^−1^)
Ningxia	270	2017	188	0
		2018	376	2,370
		2019	376	14,520
		2020	500	26,790
Yunnan	210	2017	188	0
		2018	376	5,040
		2019	376	21,300
		2020	500	30,150
Shandong	220	2018	188	0
		2019	376	4,500
		2020	376	22,500

## Discussion

CRFs are fertilizers that retain nutrients for a longer time or supplies nutrients to crops for longer periods than other fertilizers. The use of CRFs is an effective way to solve the problems of low fertilizer efficiency and environmental pollution caused by chemical fertilizers. However, the production cost of CRFs is an important factor limiting their application. In recent years, our research found that BCRF has great advantages as a low-cost, environmentally, and efficient fertilizer in orchards.

BCRF has been applied in Chinese peach orchards for 5–8 years, and we found that the application of BCRF in peach orchards in major producing areas of China has great potential to reduce nitrogen application. Compared with common fertilizer application methods (the application of common composite fertilizer), the use of BCRF can reduce the amount of nitrogen fertilizers used by 69.1–81.7, 69.0–78.4, and 65.3–76.2% for early-maturing, medium-maturing, and late-maturing peach varieties, respectively (Xiao et al., 2019). In this study, we found that peach orchards with BCRF applied in Yunnan, Ningxia, and Shandong provinces did not show a decrease in fruit yield, but the fertilizer input was significantly lower than those of common orchards. The fertilizer-saving mechanism of BCRF is analyzed below.

### Promoting the Occurrence of New Roots and Decreasing the Root Turnover Rate

The root is an important organ for crop absorption of soil water and nutrients. The nutrient type and mode of nutrient supply affect the nutrient content and distribution in soil and then significantly affect the growth, morphology, and distribution of the root system (Beare et al., 1994). The growth and development of roots directly affects the absorption of soil nutrients and soil water by roots and the growth and development of trees. In the present study, BCRF II promoted the production of new roots and increased the proportion of fine roots (Figures 9, 11). Roots grew toward fertilizers. When soil nutrients are not distributed evenly, the root will grow toward the place with abundant nutrients. The release rate of BCRF is directly related to the nutrient concentration in the soil. As nutrients are absorbed and utilized by trees, nutrients in fertilizers are released slowly, effectively reducing the loss of nutrients in soil due to rain and irrigation and ensuring the stability of the soil nutrient content. Soil nutrients under the BCRF II treatment were more stable, and nutrient movement with soil moisture was small. Therefore, the nutrients were concentrated, which was conducive to the formation of a dense root system. Fine roots are the main organs for plants to absorb nutrients and water. In our study, we also found that the fine roots of BCRF II accounted for a large proportion, accounting for 83.95% of the total root length, which was conducive to the absorption of soil nutrients by peach trees and the improvement of fertilizer utilization efficiency.

Previous study (Zhang, 2016) found that fine roots of peach trees were dense around controlled-release bags, and BCRF could not only promote the occurrence of fine roots, but also prolong the browning time of roots. In this study, the roots began to brown under FSA II treatment, but there were still more new roots under BCRF II treatment, and the active white roots were significantly more than those under FSA II treatment in September 15 (Figure 10). The result of pot experiment also showed that the root activity of BCRF I treatment was 17.53% higher than that of FSA I treatment before defoliation (Figure 5). These indicated that BCRF II slowed down the death of roots and increased the longevity of roots.

Fine root turnover refers to the generation and growth of new roots and the death and decomposition of old roots. In this study, the fine root turnover rates of peach trees were within the range of 0.127–0.179, and BCRF decreased the turnover rate of fine roots. The significant difference in turnover rates between the two fertilization modes was possibly because of the nutrient release pattern. Fine roots absorb and store nutrients through their very large surface areas, so their turnover enables the plant to utilize nutrients cyclically. Fine root turnover in plants is a reaction to reduce their energy consumption. It can meet the nutrient requirements of plants with low fine root biomass and ensure the effective absorption of soil nutrients and water (Eissentat, 1994). In this study, BCRF reduced the fine root turnover rate, indicating that the metabolism of the root during death slowed down, which increased the life span of the root, reduced the consumption for root renewal, and in turn promoted the growth of the aboveground parts.

### Improving Storage Nutrition and Ensuring Balanced Tree Growth

BCRF could provide stable nutrients for peach trees in autumn, which improved leaf photosynthesis, delayed leaf senescence, and increased root activity (Figures 1–3). On the one hand, this ensured the yield and quality of late-ripening peaches. On the other hand, it increased the accumulation of carbon and nitrogen nutrients, so that more carbon and nitrogen nutrients returned to the tree body through the leaves during the nutrient reflux period and were stored by the tree body. The present findings showed that BCRF could increase the storage nutrient level of peach trees. The contents of soluble sugar, starch, soluble protein, and free amino acids in peach trees treated with BCRF I were 15.2, 12.6, 19.2, and 28.7% higher than those in trees treated with FSA I, respectively (Figures 9, 10).

The uptake of nutrients by trees in the period from rapid expansion to ripening is at its peak, and nitrogen absorption in this stage accounts for approximately 60% of the total annual nitrogen uptake (Lobit et al., 2001). During the 2 months before the falling leaf stage, nutrients are transferred to perennial storage organs and stored in organic forms. Soluble sugar is mainly distributed in branches before leaves fall and is stored in roots during the dormant period (Hodge, 2006; Jordan et al., 2009). Vegetative storage protein (VSP) is the major form of nitrogen in fruit trees during the period of dormancy. In total, 50–70% of proteins in leaves are degraded into amino acids, which are transferred to the root cortex *via* the xylem and are reconstituted into proteins for storage (Tagliavini and Marangoni, 2002; Vanninen and Makela, 2005).

Nitrogen, as an essential nutrient element in fruit trees, has a direct impact on the development of fruit organs and the formation of tree structures (Gu, 1981). A ^15^N tracer study showed that 93% of the nitrogen needed for peach tree growth in spring was supplied from storage nutrients (Muñoz et al., 1993). Similar results were also reported in apple trees, where most of the N demand by new growth at bloom was provided by reserve N that had been remobilized from perennial parts of the trees (Cheng and Raba, 2009). In this experiment, we observed that BCRF application for 5 years resulted in healthy peach trees and stable tree structure. The reason for this is that BCRF maintains relatively stable nutrient levels in soil for many years, which provides a stable nutrient supply for peach tree growth and development. This not only reduces the stimulation of fruit tree growth by fertilization and reduces the number of secondary branches (Table 2) but also increases the accumulation of storage nutrients, which is conducive to the growth of trees in the next spring. Thus, continuous application of BCRF ensures balanced growth of peach trees.

### Reducing Ammonia Volatilization and Improving Nitrogen Use Efficiency

Ammonia volatilization is an important path for the gaseous loss of nitrogen fertilizer. In peaches, the ammonia volatilization loss under FAS I treatment accounted for 13.76% of the applied nitrogen, while BCRF I treatment resulted in the loss of 6.28%. Therefore, compared to FAS I treatment, BCRF II treatment significantly reduced ammonia volatilization loss. Similar results were also obtained under simulated field conditions, where BCRF could significantly delay the emission peak and reduce the cumulative amount of ammonia volatilization (Wang et al., 2013). After general chemical fertilizer was applied to an orchard, it came into direct contact with the soil and dissolved quickly, which increased the nitrogen concentration in the soil. As a result, NH_3_ volatilization and loss occurred in a very short period of time. However, the nutrients in BCRF were released slowly through the microholes in the bag, which kept the nitrogen concentration in the soil at a stable low level and made the NH_3_ volatilization relatively gentle. Therefore, BCRF could reduce the nitrogen gas loss, which is conducive to the cleaner production of orchards and improvement of the fertilizer utilization rate.

The nutrient utilization rate for fertilizer is an important index to measure fertilizer efficiency. Increasing fertilizer input without improving the fertilizer utilization rate threatens agricultural productivity and food security. In production, applying a high amount of fertilizer twice in double ditches is a widespread fertilization method used by farmers. A previous study suggested that the N utilization rate for this method of fertilization was reduced by 44.34% compared with that for BCRF (Xiao et al., 2019). In the present study, BCRF II treatment also increased the yield of individual plants by 21.35% on average compared to the yields of plants receiving the same amount of fertilizer by spreading (FSA II). The N utilization rate in BCRF II plants was 1.39 times that of FSA II plants. Similar results were also obtained in apple. Xiao et al. (2019) reported that BCRF reduced the amount of nitrogen fertilizer applied by 65–82% compared to that of common fertilizer application methods without decreasing peach yield.

Based on the demand for nutrients by peach trees in different growth stages, we analyzed the reasons for the improvement of the N utilization rate and yield by BCRF. First, BCRF can maintain the nutrient supply in the soil after autumn, which increases the storage nutrition in the current year and further facilitates flower bud differentiation and the construction of new organs in the following year. Second, the nutrient release from FSA and the nutrient demand of peaches were not synchronized, so ammonia volatilization and leaching loss of fertilizer were serious. This causes environmental pollution and leads to insufficient nitrogen supply in soil at the late growth stages of peaches. However, BCRF increases the number of fine roots and prolongs their survival time, thus enhancing the absorption and utilization of nitrogen. In addition, nitrogen has good mobility, and the nitrogen absorbed in the current year mainly supplies the central organs for growth (Li et al., 2010). When the fruit was the growth center, the increased level of nutrients in soil treated with BCRF enhanced the utilization of nitrogen by the fruit. This improved the N utilization rate during the fruit growth period and was beneficial to fruit expansion.

## Conclusion

Application of BCRF can promote the occurrence of fine roots and decrease the root annual turnover rate in peach trees, and it also improves the utilization efficiency of fertilizer, reduces ammonia volatilization, delays leaf senescence, and enhances storage nutrition, fruit yield, and fruit quality in peach trees.

## Data Availability Statement

The original contributions presented in the study are included in the article/supplementary material, further inquiries can be directed to the corresponding authors.

## Author Contributions

FP, YX, and YZ conceived and designed the research. YZ and JL performed and analyzed the experiments. YZ wrote the manuscript. FP and YX supervised the study and revised the manuscript. All authors read and approved the final version of the manuscript.

### Conflict of Interest

The authors declare that the research was conducted in the absence of any commercial or financial relationships that could be construed as a potential conflict of interest.
